# Effects of Long-Term Calorie Restriction and Physical Activity on Body Weight and Metabolism Applied to Different Degrees of Obesity

**DOI:** 10.5152/eurasianjmed.2026.251020

**Published:** 2026-01-01

**Authors:** Gülşah Alyar, Fatma Zühal Umudum

**Affiliations:** 1 Atatürk University Vocational School of Health Services, Erzurum, Türkiye; 2 Department of Medical Biochemistry, Atatürk University Faculty of Medicine, Erzurum, Türkiye

**Keywords:** Calorie restriction, metabolism, obesity, physical movement

## Abstract

**Background:**

The aim of the study is to investigate whether long-term calorie restriction and physical activity have an effect on body weight and metabolism in obese individuals with different body mass indexes (BMIs).

**Methods:**

Fifty-four obese women who were not receiving any obesity treatment were included in the study. Participants received 12 weeks of low-calorie diet and physical activity training appropriate to their age, gender, and BMI. Body weights were measured at the initial visit and after the intervention. Venous blood samples were collected twice: before and after the intervention. Baseline glucose, insulin, total cholesterol (TC), triglyceride (TG), HbA1c, high-density lipoprotein cholesterol (HDL-C), and low-density lipoprotein cholesterol (LDL-C) levels were measured using a Beckman Coulter AU 5800 clinical chemistry autoanalyzer. Hemoglobin A1c (HbA1c) levels were determined using boronate affinity and HPLC techniques using a Tirinity Biotech Premier 9210 autoanalyzer.Homeostatic model assessment of insulin resistance (HOMA-IR) was calculated using the formula (fasting insulin [μU/mL] × fasting glucose [mg/dL])/405).

**Results:**

In the study, insulin and HOMA-IR levels increased significantly as the degree of obesity increased (*P* < .05). Obese individuals lost an average of 8.6% weight after the combined treatment, and when repeated measurement results were compared, insulin levels in all obese groups decreased significantly (*P* < .05). HbA1c levels decreased significantly only in moderately obese individuals after the treatment (*P* < .05). In the first group (those who lost between 5% and 9.9% of their weight), TG and insulin levels decreased while TC, LDL-C, and glucose levels increased (*P* < .05). In the second group (those who lost more than 10% of their weight), only insulin levels decreased (*P* < .05).

**Conclusion:**

As obesity increased HOMA-IR levels an indicator of insulin resistance increased significantly. Participants lost an average of 8.6% of their weight and insulin levels decreased significantly after the intervention. Long-term lifestyle interventions produced relatively similar effects on metabolism across different obesity levels.

**Main Points**

- The study analyzes metabolic changes (glucose, insulin, HOMA-IR, and lipid profile) in obese individuals with varying degrees of obesity.
- The first study finding is that insulin resistance and HOMA-IR increase as the degree of obesity increases.
- The contribution of calorie restriction to metabolic changes and its interaction with physical activity were analyzed.
- The study includes a treatment protocol that evaluates the contribution of 12weeks of (long-term) calorie restriction combined with physical activity on metabolism.
- The motivation and lifestyle change compliance of individuals were evaluated after the intervention.
- The treatment program determined that all obese individuals lost an average of 8.6% of their weight.
- After the combined treatment, insulin levels were significantly reduced.
- Based on the data obtained, long-term lifestyle interventions across different degrees of obesity produced relatively similar effects on metabolism.

## Introduction

Obesity, which can occur at all stages of life worldwide, is a significant and increasingly prevalent public health problem.^1^ The disease is associated with numerous comorbidities, particularly cardiovascular and metabolic, and increases healthcare workload and costs.^2^ The primary cause of obesity, which develops multifactorially, is an energy imbalance favoring intake.^3^ In chronically positive balance excess energy is stored as high-energy molecules in fat cells, increasing their number and volume. Altered adipose tissue leads to the unregulated secretion of free fatty acids (FFA) and adipocytokines.^4^ Abundant FFAs released into the circulation reduce insulin's glucose uptake in muscle and liver cells, thereby increasing blood sugar. Unregulated adipocytokine production in turn leads to decreased insulin sensitivity and contributes to the development of chronic, low-grade inflammation.^5^ Increased release of cytokines, particularly tumor necrosis factor-alpha and interleukin-6, weakens insulin signaling by altering insulin receptor substrate-1 (IRS-1) phosphorylation.^6^ Consequently, the insulin receptor signaling pathway is disrupted, leading to insulin resistance. Because adipocytes cannot suppress lipolysis in insulin resistance, increased FFA release stimulates TG synthesis in the liver.^7^ Triglyceride (TG) is packaged in very low-density lipoprotein (VLDL) and released into the circulation. Very low-density lipoprotein exchanges cholesterol and TG with high-density lipoprotein cholesterol (HDL-C). The rapid breakdown of unstable HDL-C, enriched with TG, reduces its levels. As VLDL is broken down it transforms into intermediate-density lipoprotein (IDL) and low-density lipoprotein cholesterol (LDL-C), increasing the number of LDL particles, becoming small, dense, and prone to oxidation. These LDL particles predispose to cardiovascular disease (CVD) through the formation of atherosclerotic plaques.^8^ Consequently, metabolic dysfunction in obesity, beginning with insulin resistance, progresses to MetS, prediabetes, and subsequently CVD and type 2 diabetes mellitus (T2DM) may develop. Regular screening of obese individuals and, if necessary, treatment is crucial for preventing the development of secondary diseases.^9^ The first-line treatment options for reducing the risk of obesity and related secondary diseases are lifestyle changes that include calorie restriction and increased physical activity.^10^ These approaches, when applied independently or in combination, induce weight loss by creating a negative energy balance and improve cardiometabolic risk factors.^11^ Recent studies have demonstrated that these approaches, applied independently and in combination, have demonstrated a positive effect on cardiometabolic risk factors compared to either treatment alone.^12^^,^^13^ This study aims to investigate the effects of long-term calorie restriction and physical activity on body weight and cardiometabolic risk factors in obese individuals with varying body mass indices (BMI).

## Material and Methods

This study was conducted in accordance with the principles of the Declaration of Helsinki, after being approved by the Atatürk University Clinical Research Ethics Committee (December 29, 2021 and B.30.2.ATA.0.01.00/31) and written consent was obtained from all patients included in the study. Power analysis was used to calculate the sample size based on the data obtained by Cohen J.^14^ In order to detect a difference of 0.95 as expected, at least 14 participants in each group had to be 90% statistically significant. In this retrospective study, 18 mildly obese (BMI: 30-34.9 kg/m²), 18 moderately obese (BMI: 35-39.9 kg/m²), and 18 morbidly obese (BMI ≥ 40) volunteers who applied to the obesity center and were regularly followed up were included. The inclusion criteria for the participants were as follows: 18-60 years of age, no chronic disease, no condition preventing exercise, not receiving obesity treatment, and those who signed the informed consent form. Exclusion criteria for the study were those with major organ dysfunction, pregnant and lactating women. Those with a BMI below 18.5, and those under 18 years of age. Participants’ height and weight were measured by a trained nurse using a wall-mounted height meter and a digital scale. A 12-week calorie restriction (1000-1500 kcal/day) and physical activity (at least 5000 steps/day) program appropriate for the age, gender, basal metabolic rate, and BMI of the individuals was determined by the dietician at the obesity center. Obese individuals’ body weights were measured before and after the treatment protocols. Participants’ blood samples were taken twice in total at their first application and after the treatment. Samples were stored in a deep freezer (Nüve DF 290, Türkiye) at −80 °C. Frozen samples were thawed gradually at −20°C, +4°C, and room temperature starting from the day before the analysis. Commercial kits produced by Beckman Coulter based on a series of enzymatic reactions were used to determine the basal glucose, insulin, TC (total cholesterol), TG, HDL-C, and LDL-C levels of the samples in the Beckman Coulter AU 5800 clinical chemistry autoanalyzer. HbA1c levels were determined using boronate affinity and HPLC techniques using a Tirinity Biotech Premier 9210 autoanalyzer. Homeostatic model assessment of insulin resistance (HOMA-IR) was calculated using the formula (fasting insulin [μU/mL] × fasting glucose [mg/dL])/405.

### Statistical Analysis

The data obtained from the study were analyzed using Analyse-it Software (Analyse-it Software Ltd. Leeds, United Kingdom) and SPSS 23.0 (IBM SPSS Corp.; Armonk, NY, USA) package program. The Kolmogorov–Smirnov test was used to evaluate the conformity of the data distribution to normal distribution. Since the data did not conform to normal distribution, the Mann–Whitney *U*-test a nonparametric test, was used. The Kruskal-Wallis test was used in multiple group comparisons. Paired samples *t*-test and Wilcoxon test were used in the analysis of data from dependent groups.

## Results

Table 1 presents demographic information, laboratory values, and significance levels (*P* values) for the obese groups divided into 3 groups based on BMI. The mean age of the 54 obese women was 45.5 years (range, 38-55). When biochemical parameters were compared between the obese groups, insulin and HOMA-IR levels were found to be significantly increased (*P *= .002, *P* = .0001). Table 2 presents the biochemical results of pre- and post-treatment samples for each obesity class. When repeated measurements were compared, insulin levels were found to be significantly decreased in all obese groups. Only the moderately obese group showed a significant decrease in HbA1c and insulin levels after treatment (*P* = .042, *P* = .028, respectively). Although a decrease in TG levels was observed after treatment in the mildly and moderately obese groups, no significant difference was found (*P* > .05). In the morbidly obese group, HDL-C levels increased while TC and LDL-C levels decreased, but these did not change significantly. Only TG levels decreased significantly in this group (*P *= .003). With the 12-week calorie restriction and physical activity program, it was observed that all obese individuals lost an average of 8.4% weight. Clinically significant weight loss (> 5% weight loss) was taken as the reference and divided into 2 groups: those who lost 5-9.9% and those who lost more than 10%. The biochemical parameters of both groups at the beginning and after 12 weeks are compared in Table 3. In the first group where weight loss ranged from 5% to 9.9%, significant increases were observed in TC, LDL-C, and glucose levels while insulin and TG levels decreased (*P* < .05). No significant change was observed in HDL-C levels (*P* > .05). In the second group, where the weight loss was ≥10%, a decrease was observed in TC, LDL-C, and TG levels and an increase in HDL-C levels, but these changes were not significant (*P* > .05).

## Discussion

The study analyzed the effects of 12 weeks of calorie restriction and physical activity on metabolic changes in obese individuals grouped by BMI. When the biochemical parameters of the groups were evaluated, insulin levels were found to increase significantly as BMI increased, indicating that increased adipose tissue in obesity may reduce insulin sensitivity, leading to hyperinsulinemia and consequently insulin resistance. Furthermore, insulin and HOMA-IR levels increased significantly as the degree of obesity increased, and HOMA-IR levels in morbidly obese individuals, in particular, indicated insulin resistance in this group. Similarly, Li and colleagues^6^ argued that there is a strong correlation between HOMA-IR and BMI.Visceral fat, which increases with the degree of obesity, reduces cells’ sensitivity to insulin, impairing glucose uptake and increasing HOMA-IR values. Insulin resistance is one of the most common clinical conditions in morbidly obese individuals, and it significantly increases the likelihood of developing diseases such as T2DM, metabolic syndrome, and non-alcoholic fatty liver disease.^12^ Ba et al.^8^ found that HOMA-IR levels significantly increased in obese individuals as they transitioned from a healthy state to a T2DM state. However, when the lipid profiles of the groups were evaluated, the parameters were observed to be within the reference range. This was assumed to be due to the fact that the obese participants included in the study had no chronic diseases and the relatively short follow-up period of the study. In general, diet and physical activity are nonpharmacological approaches used to reduce the risk of weight loss and obesity-related secondary diseases.^15^ These approaches provide metabolic improvements through clinically significant (>5% weight loss) weight loss per year.^16^In this study, it was found that all obese individuals lost an average of 8.6% weight after the 12-week combined program. The results reported here are supported by a 12-month study designed for obese adults with two groups: exercise plus weight maintenance and exercise plus calorie restriction. In the same study, participants in the exercise plus calorie restriction group showed greater reductions in body weight (4.1%) and fat mass (2.6 kg) compared to exercise alone.^16^ However, this study found that insulin levels decreased significantly in all groups after combined treatment. HbA1c levels decreased significantly in moderately obese patients, and TG levels decreased significantly in morbidly obese patients. This result reiterates the importance of these treatment protocols across all degrees of obesity, especially in severe obesity. It has been argued that long-term lifestyle interventions can reduce cardiometabolic risk factors by improving insulin sensitivity. Generally, studies have found that these treatment protocols significantly reduce body weight, glucose, HbA1c, and HOMA-IR levels in overweight or obese adults with T2DM.^11^^,^^12^^,^^15^

Another study objective was to evaluate the relationship between percent weight loss and cardiometabolic risk factors. Obese individuals with weight loss between 5% and 9.9% (group 1) and obese individuals with weight loss greater than 10% (group 2) were classified. In the first group, TC, LDL-C, and glucose values increased significantly after treatment, while TG and insulin levels decreased. The decrease in TG and insulin levels suggests that lifestyle changes have a positive impact on metabolism. While a decrease in LDL-C and TC is generally expected with weight loss, the increase in these values suggests that altered body fat mobilization during weight loss may cause temporary increases in plasma lipids. In the second group, only insulin levels decreased significantly after combined treatment. Furthermore, TC, LDL-C, and TG levels decreased while HDL-C levels increased, but these changes were not significant. The trend toward a decrease in TC, LDL-C, and TG in these parameters suggests that further weight loss has a positive effect on the lipid profile. However, the fact that these effects were not significant reveals the need for more careful evaluation of sample size, follow-up duration, and individual differences. Contrary to this study, Jung et al.^17^ determined that a 12-week calorie restriction and exercise program applied to obese patients provided a significant decrease in BMI, TG, and TC levels. Jorge et al^18^ found significant decreases in basal glucose, postprandial glucose, and lipid metabolism after 12 weeks of different exercise programs (aerobic exercise, resistance exercise, combined (aerobic + resistance)) in obese patients with T2DM.

A study in obese adults including an exercise intervention alone, dietary modification plus body weight maintenance, and dietary modification plus energy restriction (weight loss) found that all participants lost approximately 4% of their body weight.The study also reported significant improvements in glucose and HDL-C levels in the weight-loss group compared to the exercise group.^13^ In a 12-month study conducted with 10  439 overweight or obese postmenopausal women, Foster-Schubert and colleague^19^ divided participants into 4 groups: control, diet alone, exercise alone, and diet plus exercise. Compared to the control group, the diet group lost 7.1 kg, the exercise group 2.0 kg, and the diet plus exercise group 8.9 kg. Therefore, the combination of diet and exercise resulted in a greater weight loss (1.8 kg) compared to diet alone, and the difference was statistically significant. Consequently, the weight loss achieved when calorie restriction and physical activity protocols were combined was greater than that observed with diet alone. Furthermore, studies using diet plus exercise protocols together reported similar results, with weight loss of at least 3-5% or more.^12^^20^

As obesity increased HOMA-IR levels an indicator of insulin resistance increased significantly. Participants lost an average of 8.6% of their weight and insulin levels decreased significantly after the intervention. However, long-term lifestyle interventions produced relatively similar effects on metabolism across different obesity levels.

## Figures and Tables

**Table 1.** Comparison of Demographic Variables and Laboratory Values of Obese Individuals Divided into 3 Groups According to Body Mass Index Values

	**Mildly Obese **	**Moderately Obese**	**Morbidly Obese **	***P***
**BMI**	32.8 (32.2-34)	38.7 (36.4-39.2)	46 (42.7-49)	.0001*
**Age (year)**	44.5 (39-51)	45 (34-54)	48 (40.4-56)	.426
TC (mg/dL)	207.8 (180.4-235)	183.7 (164-229)	195.2 (167.8-220)	.339
HDL-C (mg/dL)	48.5 (42.1-58.3)	46.1 (36.5-50)	46.2 (42-53.2)	.371
**LDL-C (mg/dL)**	124.5 (105-151)	109 (92-137)	114 (92.5-129.5)	.225
TG (mg/dL)	143.5 (84-177.8)	147 (97-190.7)	131.5 (103-176.5)	.865
Glucose(mg/dL)	89 (84-96)	92 (84.2-107.7)	90 (82.5-104)	.596
HbA1c (%)	5.7 (5.5-5.9)	5.9 (5.6-6.2)	5.9 (5.5-6.1)	.358
Insulin (µU/mL)	8.5 (5.7-10.6)	10.5 (6.7-18.3)	15.4 (10.6-19.1)	.002*
HOMA-IR	1.87 (1.18 – 2.51)	2.39 (1.39-4.87)	3.42 (2.16-4.91)	.0001*

HbA1c, hemoglobin A1c; HDL-C, high-density lipoprotein cholesterol; HOMA-IR, Homeostatic model assessment of insulin resistance; LDL-C, low-density lipoprotein cholesterol; TC, total cholesterol; TG, triglyceride.

**Table 2.** Comparison of Pre- and Post-Treatment Measurements in Obesity Degrees

		**1. Measurement** **Median (IQR) **	**2. Measurement** **Median (IQR)**	***P***
**Mildly Obese**	**TC (mg/dL) **	207.8 (180.4-235)	225.5 (188.8-255.8)	.303
	**HDL-C (mg/dL)**	49 (42.7-58.3)	47 (41.1-59.3)	.345
	**LDL-C (mg/dL)**	125 (105-150)	142 (118.2-162)	.087
	**TG (mg/dL) **	147 (89.3-178.3)	120 (83.3-176.1)	.275
	**Glucose (mg/dL) **	88 (84.3-95.6)	95 (86.1-97)	.181
	**HbA1c (%) ) **	5.7 (5.5-5.9)	5.7 (5.6-5.9)	.782
	**Insulin (µU/mL)**	8.5 (5.7-10.6)	7.1 (5.2-9.4)	.031*
	**HOMA-IR**	1.85 (1.19-2.50)	1.67 (1.11 – 2.25)	.1674
**Moderately Obese**	**TC (mg/dL)**	183.7 (164-229)	201 (157.2-263.3)	.123
	**HDL-C (mg/dL)**	46 (36-50)	43 (38.5-54.2)	.644
	**LDL-C (mg/dL)**	109 (92-138.7)	129 (92.5-171.7)	.054
	**TG (mg/dL) **	147 (95.5-190.8)	133 (101.3-175.5)	.062
	**Glucose (mg/dL) **	92 (84.2-108.2)	99 (85.8-106.8)	.418
	**HbA1c (%) **	5.9 (5.6-6.23)	5.7 (5.4-6.1)	.042*
	**Insulin (µU/mL)**	10.5 (6.7-18.3)	9.6 (7-14)	.028*
	**HOMA-IR **	2.39 (1.39-4.89)	2.35 (1.48–3.69)	.5166
**Morbidly Obese**	**TC (mg/dL)**	195.2 (167.8-220)	189 (1735-209.7)	.259
	**HDL-C (mg/dL)**	46.2 (42-53.2)	49 (42.4-56.2)	.388
	**LDL-C (mg/dL)**	114 (91.9-129.7)	112.5 (99.4-134.5)	.056
	**TG (mg/dL) **	131.5 (102-178.9)	121 (96.4-146.9)	.003*
	**Glucose (mg/dL) **	90 (82.4-104.2)	92 (87.2-106.5)	.063
	**HbA1c (%) **	5.9 (5.5-6.1)	5.8 (5.44-6.05)	.329
	**Insulin (µU/mL)**	15.4 (10.6-19.1)	13.3 (9.4-14.6)	.002*
	**HOMA-IR**	3.42 (2.16-4.91)	3.02 (2.02-3.84)	0.275

HbA1c, hemoglobin A1c; HDL-C, high-density lipoprotein cholesterol; HOMA-IR, Homeostatic model assessment of insulin resistance; LDL-C, low-density lipoprotein cholesterol; TC, total cholesterol; TG, triglyceride.

**Table 3.** Comparison of Repeated Measurements in Groups Created According to Weight Loss Percentages

		**1. Measurement** **Median (IQR) **	**2. Measurement** **Median (IQR)**	***P***
**Group I** **(n = 36)**	**TC (mg/dL)**	197 (169-227.9)	209.4 (176-240)	.0243^*^
	**HDL-C (mg/dL)**	47.7 (42-54.6)	48.2 (41.9-56.6)	.729
	**LDL-C (mg/dL)**	116 (96-142)	134 (99-161)	.002^*^
	**TG (mg/dL) **	144.9 (112-185)	130.6 (90-184)	.003^*^
	**Glucose (mg/dL)**	90 (84-102)	96 (86.4-106)	.016^*^
	**HbA1c (%)**	5.9 (5.6-6.2)	5.8 (5.6-6.1)	.071
	**Insulin (µU/mL)**	10.6 (7-16)	9.7 (6.2-14)	**.051**
	**HOMA-IR**	2.58 (1.4-4)	2.34 (1.3-3.6)	.658
**Group II** **(n = 18)**	**TC (mg/dL) **	197.5 (162.6-221.2)	192.1 (168.1-217.5)	.644
	**HDL-C (mg/dL) **	43.4 (40.4-50.4)	46.2 (37.9-49.7)	.761
	**LDL-C (mg/dL)**	118 (101-133)	121 (98-133)	.145
	**TG (mg/dL) **	119.7 (78.7-190.3)	112.5 (88.9-145.3)	.190
	**Glucose (mg/dL)**	89 (83.9-103.2)	90.5 (84.9-97.4)	.517
	**HbA1c (%)**	5.6 (5.19-5.91)	5.55 (5.1-5.73)	.426
	**Insulin (µU/mL)**	12.8 (10.5-16.9)	10.2 (7-12.5)	.001^*^
	**HOMA-IR **	2.81 (2.18-4.31)	2.28 (1.47-3.01)	.365

Group-1: weight loss: %5-9.9; Group-2: weight loss %≥10.

## Data Availability

The data that support the findings of this study are available on request from the corresponding author.
